# 

**Published:** 1894-07

**Authors:** 


### CONTRIBUTORS TO VOL. X.

Beall, Dr. E. J., Fort Worth ............135
Boyd, Dr. R. H. A., San Antonio........325
Brown & Bailey, Drs., Richland . .......502
Bennett, Dr. T. J., Austin ...............505
Bermingham, Dr. Edw. H., New York 589

Cook, Dr. E. T., Houston . ...............218
Coleman, Dr. P. C., Colorado City .....221
Church, Dr. B. F., Terrell ...............503
Caspar, Dr. F. S., Austin.................470
Cerna, Dr. David, Galveston, 226, 369 to 372, 448, 508
Conerly, Dr. T. W., San Angelo ........151
Cummings, Dr. J., Austin ................157
Caffery Russell, San Antonio ..........587

Dyer, Prof. Isadore, M.D., New Orleans 78, 173, 232, 288
Downs, Dr. L. S., Galveston ............350

Eaves, Dr. J. F., Millican ................585
Erichsen, Dr. Jno. Eric, London, Eng.114

Field, Dr. J. T., Fort Worth ...........163
Flavin, Dr. T., Galveston...........171, 230
Fuller, A. S., Houston. ........... ..147, 286

Grayson, Dr. T. B., Winkler............507
Gluck, Dr. Isadore, Houston ...........278
Goelet, Dr. A. H., New York ........80, 444
Gammon, Dr. Wm., Galveston .........498

Hollis, Dr. L. W., Abilene ..........394, 282
Hill, Dr. L. D., Webberville ........... 348
Hilgartner, Dr. H. L., Austin ........... 74
Hathcock, Dr. A. L., Palestine .........385

Johnson, Dr. James, Brownwood .......273
Jackson, Dr. E. B., Houston.............593

Kennedy, Dr. Jas., Galveston ...........440
Keiller, Prof. Wm., M. D., F. R. C. S., London, Eng................24, 60, 121, 511

Loeb, Prof. H. W., M. D., St. Louis ....338

Menger, Dr. R., San Antonio...........115
McLaughlin, Dr. J. W., Austin ........224
Morris, Dr. Robt. T., Houston..........213
Moss, Dr. R. E., San Antonio .......70, 267

Oates, Dr. T. F., Mexia...............11, 161
Outten, Prof. W. B., M. D., St. Louis.. 97
Orr, Dr. Jas., Terrell ......................207

Pugh, Dr. W. W., Hearne ..... ........393
Parker, Dr. Daniel, Calvert ............108
Perkins, Dr. A. N., Sabine Pass ........223

Rogers, Dr. W. N., Belton ..............174
Red, Dr. S. C., Houston..... ............343
Ryder-Taylor, Mr. H., San Antonio ...446

Souchon, Prof. Edw., New Orleans ....399
Smith, Prof. A. J., M. D., Galveston, 20, 49, 165, 118, 515
Swearingen, Dr. R. M., Austin........1, 97
Stout, Dr. S. H., A. M., L.L. D., Dallas 493
Smith, Dr. M. M., Austin................437

Thompson, Prof. J. E., M. D., F. R. C. S., Galveston............. ..... ..331, 433

Wallace, Dr. D. R., Waco ................569
Wagley, Dr. T. J., Cleburne...... ...... 15
Walker, Dr. W. W. Schulenburg ......161
White, Dr. J. W., Philadelphia ....... 390
Weir, Dr. James, Owensburg, Ky. .....405
Wooten, Dr. T. D., Austin ...............225

### CONTENTS—VOLUME X.

A Literary Bombshell .......... 459
Antipyretic, The New .......... 462
Asylum Management, Reform in .......... 309
Association, The T. S. M. .......... 249
A Specious Plea .......... 417
American Public Health Association .......... 125, 244
Appointments, The .......... 354
A Bill to Establish a Department of Public Health .......... 474
Abdominal Hysterectomy .......... 157
Another Texas Medical College .......... 134
As Others See Us .......... 192
A Great Sanitarian on Spinal Concussion .......... 193

Bill, A, to Establish a Department of Public Health .......... 474
Book Notices .....35, 137, 195, 258, 319, 373-378, 427, 478, 623
Better Organization in Medicine .......... 348
Black Jaundice .......... 84
Biographical, Dr. George Cupples .......... 605
" Dr. P. C. Coleman .......... 608
Blindness in Texas .......... 74
Bombshell, A Literary .......... 459
Brain Localization in Epilepsy .......... 455
Briggs, Professor, Death of .......... 29
Cupples, Dr. Geo., Death of .......... 605-620
Castration for Hypertrophy Prostate Gland .......... 390
Coleman, Dr. P. C., Biography of .......... 608
Cysts of Spermatic Cord and Testicle .......... 433
Cerebral Syphilis .......... 325
Concussion of the Spine, Comments on Erichsen's .......... 343
Catarrh, Chronic, of the Middle Ear, Cause, etc. .......... 267
Consumption, Pulmonary, Treatment of .......... 49
Chloroform Administration .......... 60
Confederate Veterans, The United .......... 538
Congress, The Two Bills in .......... 464
Catarrh and Its Location, etc .......... 393
Cystitis .......... 218
Croupous Rhinitis .......... 657

Diphtheria and Membraneous Croup .......... 394
Diphtheria, Serum Treatment in .......... 372
Dermatology, Notes on .......... 78, 173, 232, 288
Department of Public Health, A Bill to Establish a .......... 474
Dr. Wallace's Paper .......... 609
" " Discussion on .......... 582
Downs, Dr. L. S., Letter from .......... 350
Do the People Need Protection from Quacks .......... 668
Dysentery, The New Mexican Remedy in .......... 223

Department of Therapeutics............226, 372, 369, 448, 508
" Gynecology..............24, 151, 171, 230, 511
" Practice of Medicine........ ..20, 118, 165, 515
" Dermatology....................173, 232, 288
" Dentistry ....... ...................... 470
Dignity and Impudence.... ........................ 357
Death of Dr. F. L. Sim.................................. 369
" Prof. Briggs.................................. 49
" Dr. Cupples ................. ............... 605
Dislocation of the Knee, Outward ......... ............. 505
Deadly Oysters ......................................... 365
Discussion on Dr. Wallace's Paper ...................... 582
Dallas Meeting Texas State Medical Association .......... 595

Erichsen, Dr. Jno. Eric, Letter from..................... 114
Emasculation of Masturbators; is it Justifiable............ 239
Epilepsy, a case of........... . .................... 278
Ether Anæsthesia, Discovery of..... ................ 530
Epilepsy, Brain Localization in.......................... 455
Endo metritis............................................ 15

Great Men Differ......................................... 359
Glandular Tumors of Neck................................ 325
Gonorrhœa, Relation to Pelvic Disease.................. 587

Hysterectomy, Abdominal ............................. 157
Hygiene in the Young in School......................... 256
Hydrophobia, Was it.................................... 502
Hemorrhage, Umbilical..... ....................... 507
Hernia, Strangulated, a Case of......................... 286
Hill, Dr. L. D., Letter from............................. 348
Health Department; Texas' One Man................... 351

Is it the Beginning of the End?.... .................. 405
Increase of Mental Unsoundness....................... 569
Indecent Publications in Newspapers ................... 466
"Increase of Mental Unsoundness"—A Review of Dr. Wallace's Paper ................................ ....... 646

Journal (The) and the Association....... ............ 527

Knee, outward Dislocation of.......................... 505

Letter from Dr. Rogers on Typhoid Fever.......... ..... 174
" " Dr. Hill .................................. 348
" " Dr. Erichsen ............................. 114
" " Dr. McLaughlin........................... 224
Library Museum Medical College of Texas.... .......... 83

Morphine Narcosis, a Case of........................... 585
Mental Unsoundness, Increase of......................... 569
Malarial Hæmatinuria..................................... 11
Medical Law, the Texas....... ...................... 537
Mare's Nest, a.......................................... 561

Medical News and Miscellany . . 3, 85, 135, 250, 256, 312, 361, 368, 468, 540, 606, 670.
Mexican Remedy for Dysentery, the new . . . . . . . . . . . . . . 223
McLaughlin, Dr. J. W., Letter from . . . . . . . . . . . . . . . . 224
Medical Legislation in Texas . . . . . . . . . . . . . . . . . . . 350
Mirror Test, the, for Nasal Obstruction . . . . . . . . . . . . . 338
Medico-Legal Society, The . . . . . . . . . . . . . . . . . . . . 237
Mississippi Valley Medical Association — the Hot Springs Meeting . . . . . . . . . . . . 126, 300
Mastoid Operation, The . . . . . . . . . . . . . . . . . . . . . . 503
Medical Profession (The) and Life Insurance Companies . . . 493
Masturbators, Emasculation of . . . . . . . . . . . . . . . . . . 239
Medical Advertising and the Mails . . . . . . . . . . . . . . . . 620
Medical Law, We Need a Better . . . . . . . . . . . . . . . . . . 225
Minor Railroad Injuries, Treatment of . . . . . . . . . . . . . . 285
Malarial Hæmatinuria, Treatment of . . . . . . . . . . . . . . . 161
Miasmatic Paralylitic Fever, or Pernicious Malarial Fever . . 629

North Texas Medical Association, The . . . . . . . . . . . . . . 307
Neck, Glandular Tumors of . . . . . . . . . . . . . . . . . . . . 331
Not dreamt of in Horatio's Philosophy . . . . . . . . . . . . . . 533
New Antipyretic, the . . . . . . . . . . . . . . . . . . . . . . . 462
Newspapers, Indecent Publications in . . . . . . . . . . . . . . 464
Nasal Obstructions, Early Treatment of . . . . . . . . . . . . . 70

Obstruction, Nasal, Early Treatment of . . . . . . . . . . . . . 70
Obstruction, Nasal, Miror Test of . . . . . . . . . . . . . . . . 338
Opium Disease in Children . . . . . . . . . . . . . . . . . . . . 478
Ovarian Pathology, Traumatism in . . . . . . . . . . . . . . . . 440
Organization in Medicine . . . . . . . . . . . . . . . . . . . . . 348
Organization, Medical, in Texas . . . . . . . . . . . . . . . . . 221

Prof. Smith's Papers . . . . . . . . . . . . . . . . . . . . . . . 85
Practice of Medicine, Department of . . . . . . . . 515, 118, 165, 20
Post Partem Hemorrhage, Two Cases of . . . . . . . . . . . . . 147
Potent Drugs in the Hands of Patients., Etc . . . . . . . . . . 593
Pyorrhœa Alveolaris . . . . . . . . . . . . . . . . . . . . . . . . 470
Puerperal Eclampsia . . . . . . . . . . . . . . . . . . . . . 282, 213
Pulmonary Consumption, Treatment of . . . . . . . . . . . . . . 49
Puerperal Sepsis . . . . . . . . . . . . . . . . . . . . . . . . . . 477

Quarantine (Texas) Expenses of the . . . . . . . . . . . . . . . 353

Review of Dr. Wallace and The Railway Surgeons on Spinal Concussion . . . . . . . . . . . . 1
Rhinological Don'ts . . . . . . . . . . . . . . . . . . . . . . . . 589
Relationship of Gonorrhœa to Pelvic Disease . . . . . . . . . . 587
Retrospective, Prospective . . . . . . . . . . . . . . . . . . . . 33
Railway Spine . . . . . . . . . . . . . . . . . . . . . . . . . . . . 184
Regular Medical Profession (The) How Shall it be Organized 108
Railway Spine, Letter from Jno. Eric Erichsen . . . . . . . . . 114
Railway Injuries (Minor) Treatment of . . . . . . . . . . . . . . 285
Reductio ad Absurdum . . . . . . . . . . . . . . . . . . . . . . . 369

Reform in Asylum Management........................ 309
Rally to the Standard................................ 247
Railroad Spine....................................... 127
Remedy for Dysentery, The Mexican.................... 223
Richmond Academy of Medicine Meeting ................ 665
Report of a Few Surgical Cases ...................... 637
Recent Progress in Ophthalmology..................... 658

Spontaneous Combustion, So Called.................... 115
Surgical Work, Some, in San Antonio.................. 115
Spina Bifida, Operation, Cure........................ 163
Syphilis, Cerebral .................................. 325
Serum Treatment of Diphtheria........................ 372
Sexual Disability in Males, Increase of.............. 207
Spinial Concussion................................... 273
Strangulated Hernia, a Case of....................... 286
Sims, Dr. J. Marion, Reminiscences of................ 399
Sim, Death of Dr. F. L............................... 369
State Health Officer's Report........................ 291
Skin Grafting, Theirsh's Method in................... 498
Spermatic Cord and Testicles, Cysts of............... 432
Spinal Concussion, A Great Sanitarian on............. 193
Spinal Concussion, A Review of Dr. Wallace on........ 1
Spook, A, That will not Down......................... 27
Shall we Abolish the T. S. Medical Ass'n............. 133

Typhoid Fever, Treatment of.......................... 174
Tuberculosis, Curability, Climatology, etc........... 151
The Transactions; A Correction....................... 224
Transactions, The............................... 224, 249
Texas Quarantine Expenses............................ 353
Texas State Med. Ass'n. Dallas Meeting of............ 595
Texas State Med Ass'n................519, 525, 452, 453, 177
Texas State Med Ass'n., Shall we Abolish............. 133
That Decision........................................ 539
Treatment of Pulmonary Consumption................... 49
Treatment of Malarial Hematuria...................... 161
Treatment of Uterine Fibroids........................ 444
Treatment of Typhoid Fever........................... 174
Traumatic Neurosis, Reply to Dr. Swearingen.......... 97
Texas Medical College, Another....................... 134
Texas' One Man Health Department..................... 351
The Mexican Dysentery Remedy Again................... 663
The Journal—Close of Volume X........................ 667

Uterine Fibroids, Treatment of....................... 446

What Next?........................................... 182
Wooten Bill, The..................................... 424
We Need a Better Medical Law......................... 225
Whited Sepulchre, A.................................. 539

